# Correction to ‘Procrastination Within the Nursing Context: A Concept Analysis’

**DOI:** 10.1002/nop2.70448

**Published:** 2026-04-08

Zhang, H. X. Mai, J. Zhang, M. Zhu, Y. Liu. 2025. “Procrastination Within the Nursing Context: A Concept Analysis.” Nursing Open 12, no. 10: e70308. https://doi.org/10.1002/nop2.70308.

On page 2, paragraph 1 of the section “2.3 Search Methods and Outcomes”, the sentence “Reviews, editorials, non‐empirical reports and duplicates were excluded.” has been updated to: “Editorials, non‐empirical reports and duplicates were excluded.”

In addition, on page 3, Figure 1 (PRISMA flow chart) has been updated, and the revised figure is provided below.

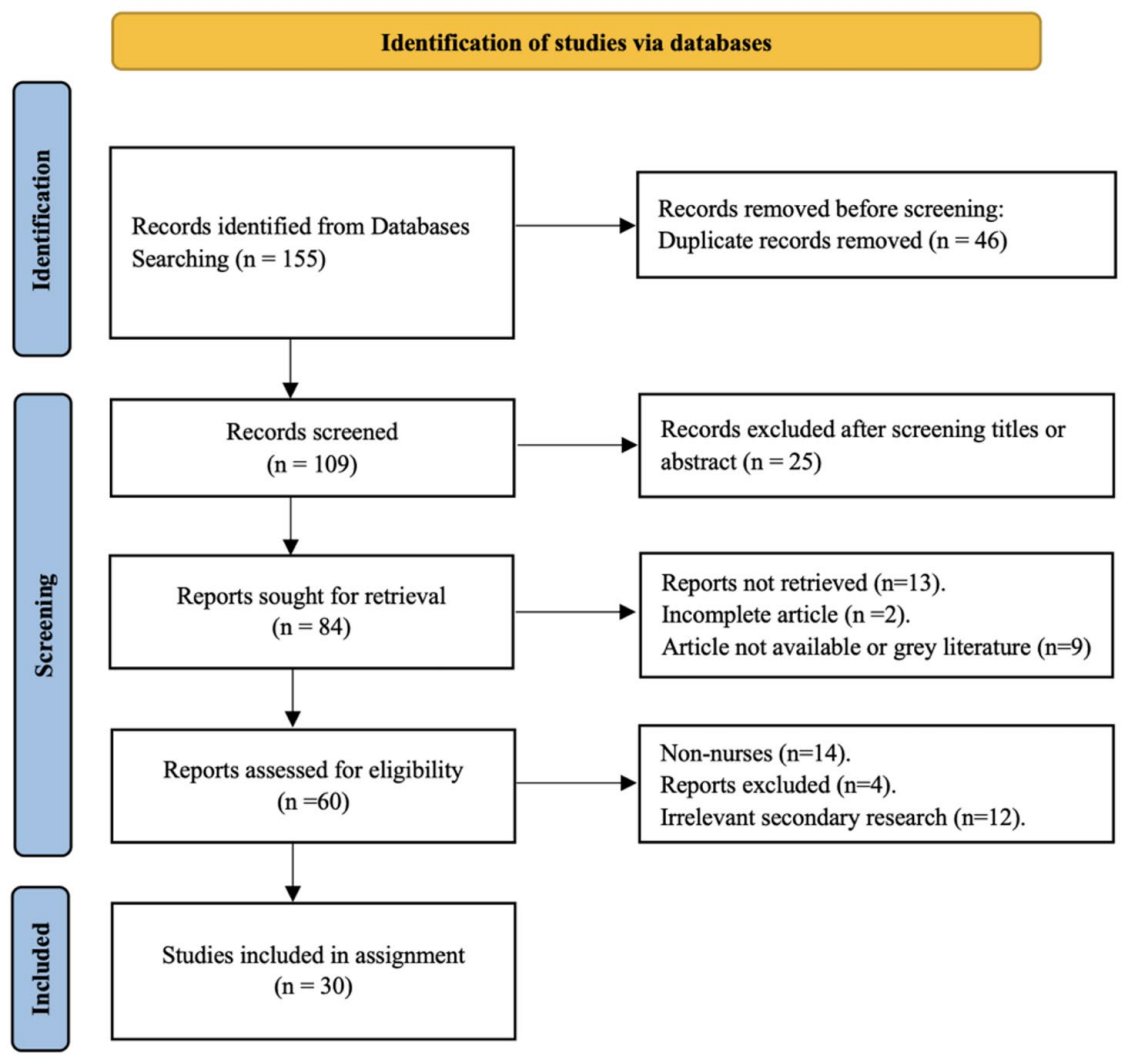

We apologize for this error.

